# Advances in immunological research on male infertility

**DOI:** 10.3389/frph.2026.1752758

**Published:** 2026-04-20

**Authors:** Zejun Liu, Xinran Yang, Yuan Ji, Shen Wang, Jingqi Wang

**Affiliations:** 1 Department of Urology, Second Hospital of Shanxi Medical University, Taiyuan, China; 2 Department of Emergency Medicine, Shanxi Provincial People’s Hospital, Taiyuan, China

**Keywords:** blood–testis barrier, immune regulation, male infertility, oxidative stress, testicular immune microenvironment

## Abstract

Male infertility is an increasingly serious global health problem. As an organ with immune privilege, the testis possesses a unique immune microenvironment that shapes normal spermatogenesis. Distinct from the testicular microenvironment, the epididymis plays a critical role in both sperm maturation and immune regulation. This review aims to systematically describe the regulatory mechanisms of the testicular and epididymal immune microenvironment and, using various diseases as examples (varicocele, testicular torsion, prostatitis, orchitis, viral infections, environmental toxicant exposure, aging and obesity), to explore in depth how disruption of this balance leads to male infertility through immunological pathways. Targeted antioxidant and anti-inflammatory interventions on specific immune pathways currently represent major therapeutic directions. Although many precision interventions and other emerging therapies remain at the experimental stage, they provide broad prospects for the development of novel treatment paradigms.

## 1 Introduction

**Figure 1:**
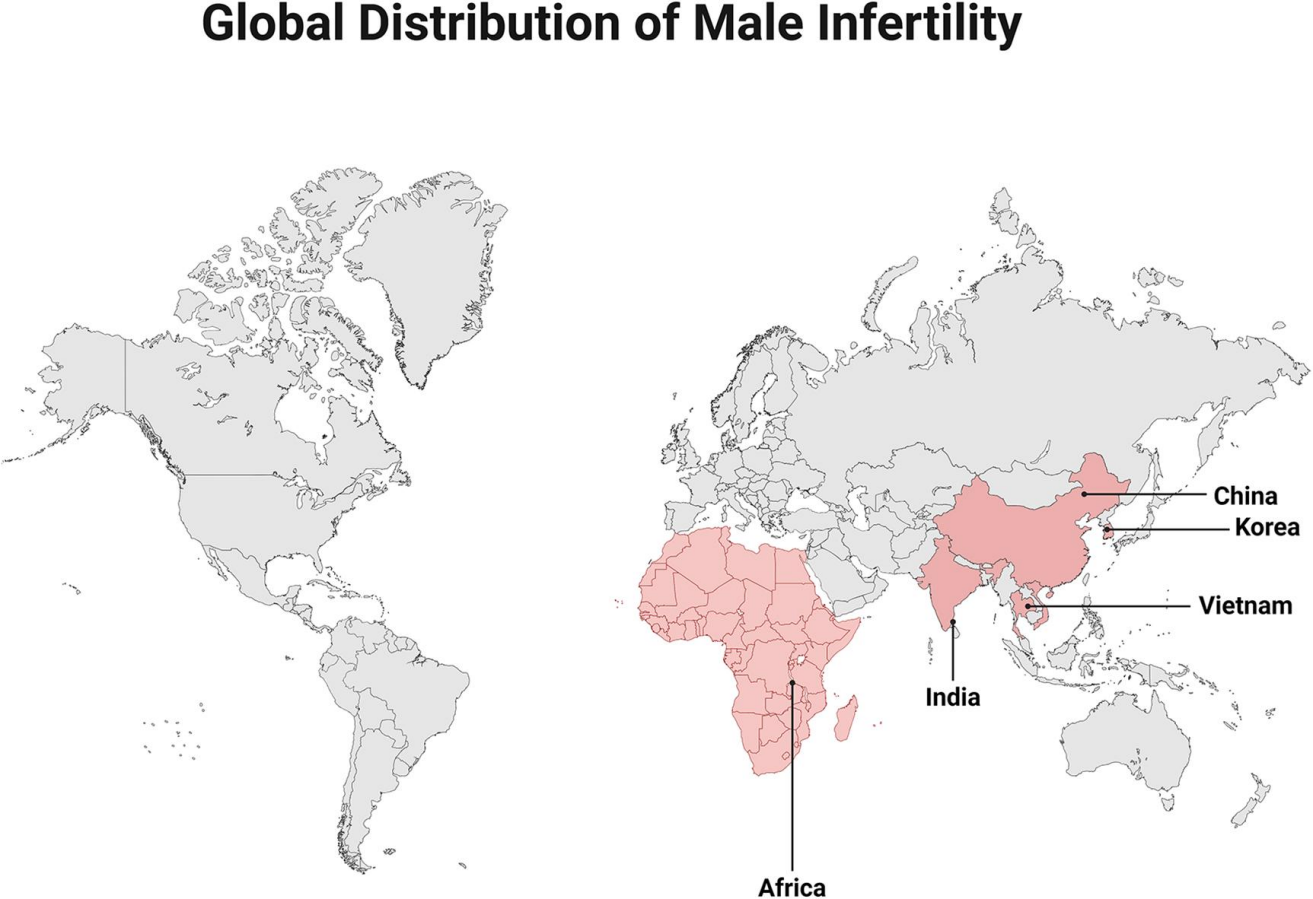
Global distribution of male infertility.

Infertility is defined as failure to achieve pregnancy after 12 months of regular unprotected intercourse, with male factors accounting for up to 50% of cases (1). A global epidemiological survey found that the incidence of male infertility increases by 0.291% per year (2).In Africa and East Asia, the prevalence of male infertility peaks in the 30–34 years age group (3) (Figure 1 illustrates the global distribution of male infertility.), and the high burden of infertility among young men has become a worldwide concern. The testis possesses a unique immune microenvironment in which local immune mediators of germ cells, immune tolerance, and the physical immune barrier blood testis barrier (BTB) jointly maintain immune privilege. Disruption of the immune system or the development of autoimmunity can destroy this privilege and result in male infertility (4). Utilizing the blood–epithelial barrier, the epididymis possesses a structural defense system nearly identical to that of the testis. The epididymal interstitium is densely populated with diverse cell types, including mesenchymal fibroblasts and immune cells. Infection and inflammation can impair sperm maturation and ultimately lead to infertility.In the management of male immunological infertility, many challenges remain, including limited accuracy of antisperm antibody testing and the uncertain efficacy yet considerable adverse effects of hormonal therapies. This review aims to delineate the immune systems of the testis and epididymis. Additionally, it provides in-depth insights into recent therapeutic advances for various associated diseases and synthesizes current treatment modalities.

## 2 Testicular immune microenvironment

**Figure 2:**
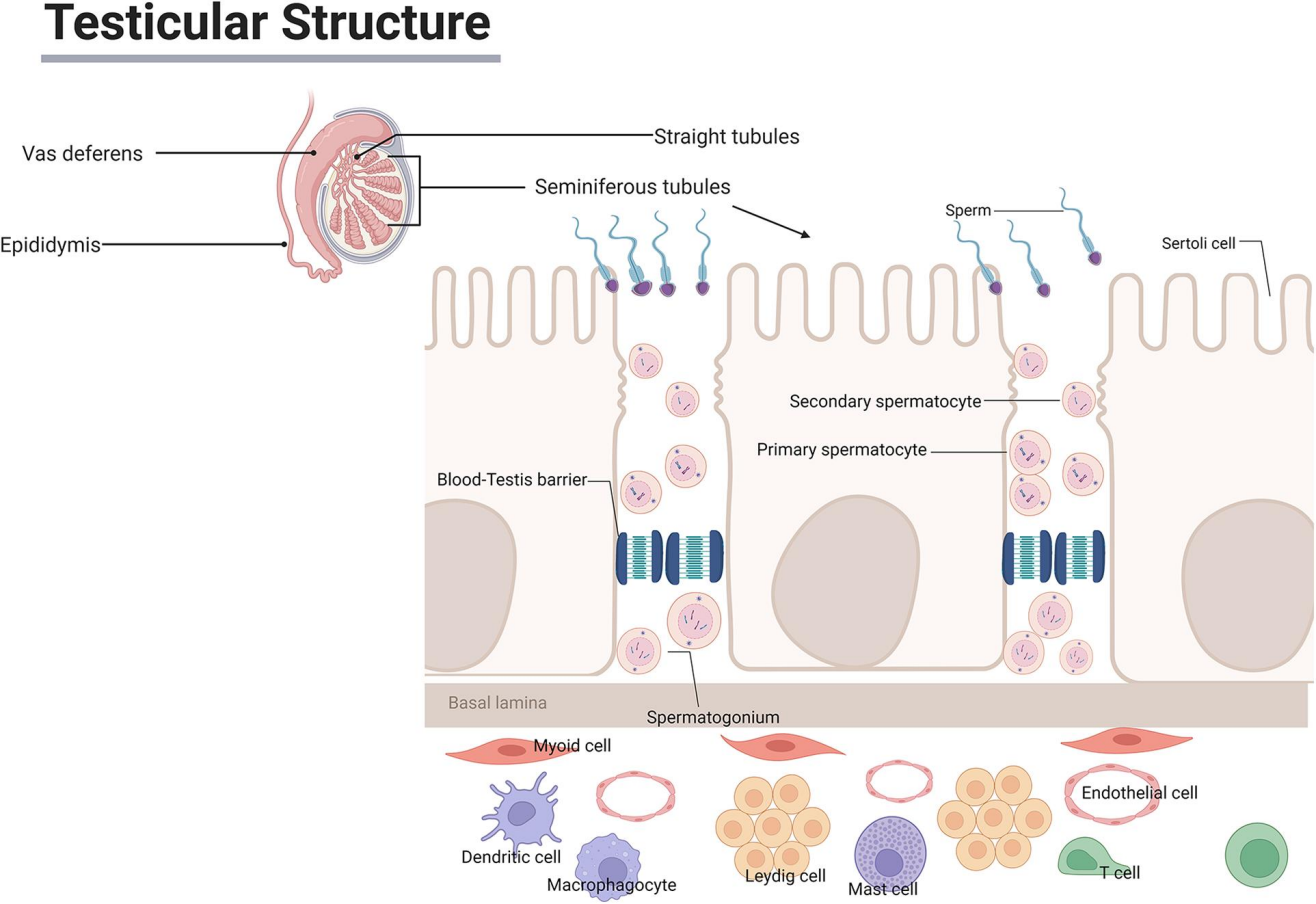
Schematic representation of the testicular immune microenvironment and the blood-testis barrier. This figure illustrates the anatomical basis of testicular immune privilege.

Spermatogenesis and sperm maturation are inextricably linked to the male testis, and a structurally normal testis is essential for maintaining male fertility and reproductive health. Spermatogenesis is a complex process that begins with undifferentiated spermatogonia, which differentiate into spermatocytes and then undergo a transition from mitosis to meiosis to generate spermatids, followed by gradual maturation into spermatozoa (5, 6). It is the maintenance of this testicular immune microenvironment that allows most men to sustain vigorous and functional germ cells. The testicular immune microenvironment primarily consists of the BTB, testicular cells, various immune cell populations, and immune mediators (Figure 2 illustrates the anatomical basis of testicular immune privilege).

### 2.1 Immunoregulatory functions of the BTB

**Figure 3:**
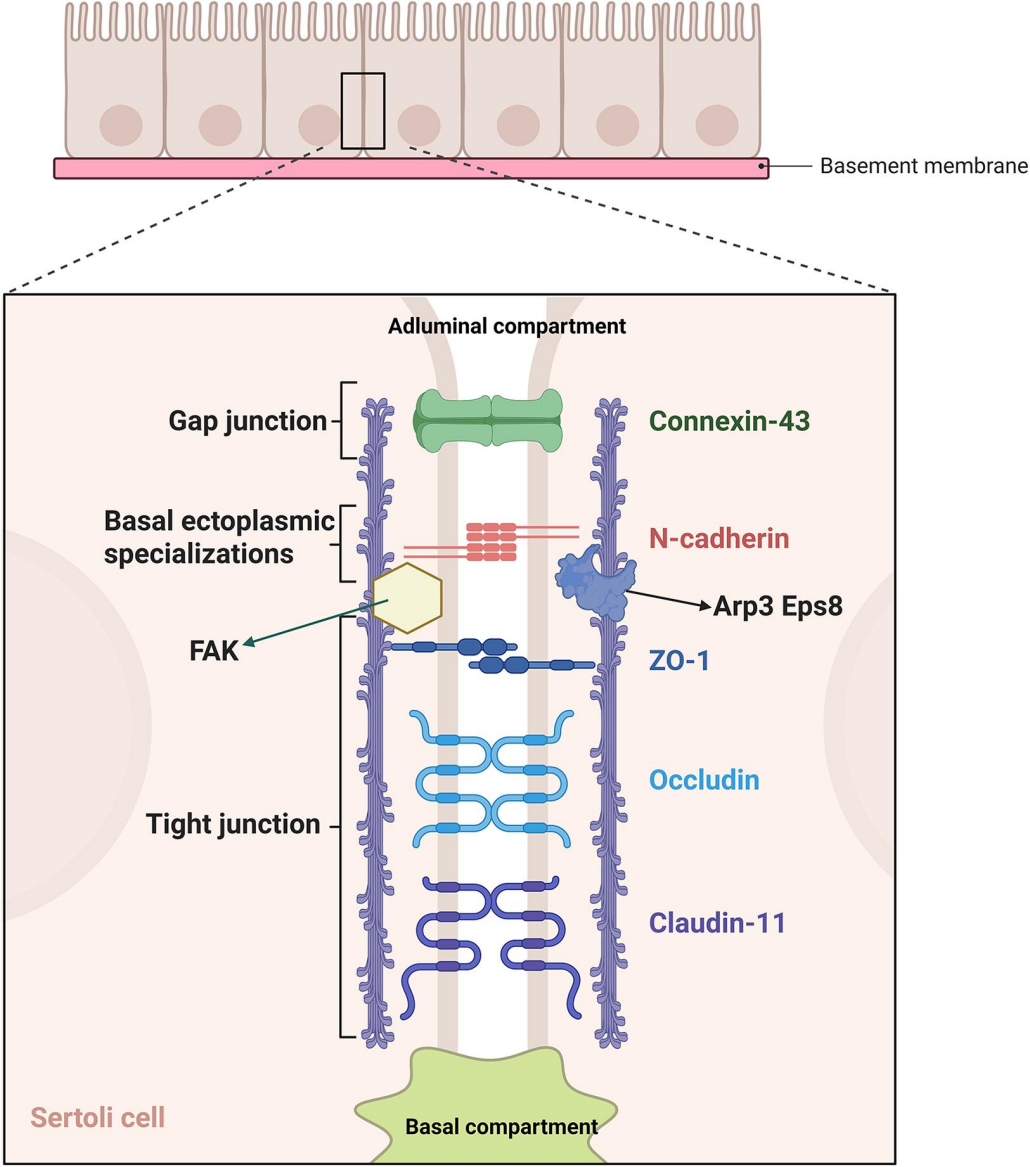
Molecular architecture of the blood-testis barrier between adjacent sertoli cells.

**Figure 4:**
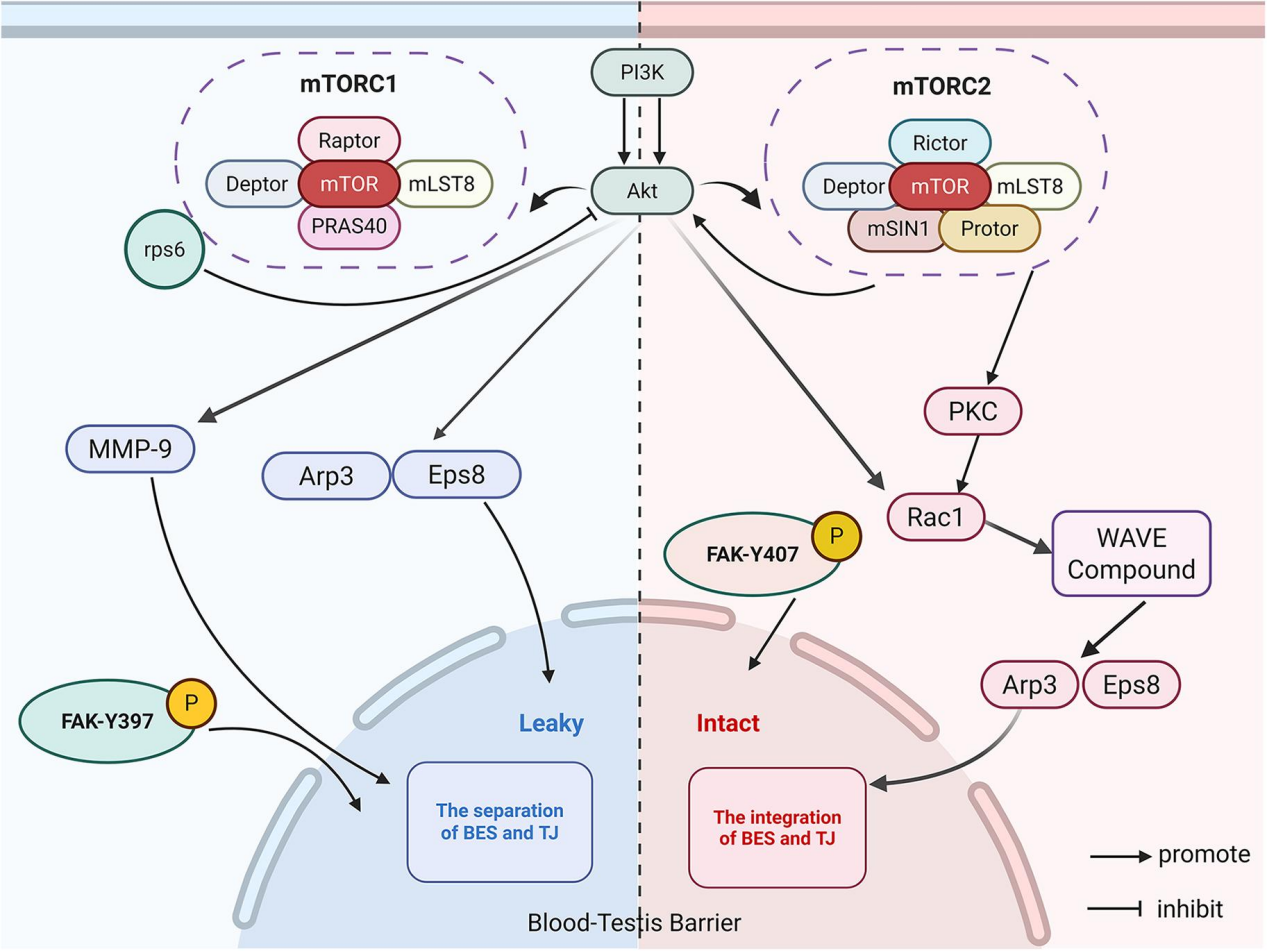
Signaling pathways regulating blood-testis barrier dynamics: the interplay between mTORC1 and mTORC2. This schematic illustrates the opposing roles of the mTOR complexes in regulating actin dynamics and junctional integrity at the BTB. Blue denotes compromised pathways, while red denotes intact pathways. BES, basal ectoplasmic specialization; TJ, tight junction.

Proteins expressed on the sperm surface can be recognized by the body as foreign antigens. To prevent autoimmunity, the testis has evolved a structural barrier BTB to safeguard male fertility (7). The BTB was initially identified in the early 1900s (8), and the development of modern microscopic techniques has greatly deepened our understanding of its structure and function (9). Structurally, the BTB resides in the seminiferous epithelium and divides cells into a basal compartment and an adluminal compartment (10). Between neighboring Sertoli cells, tight junctions(TJs), gap junctions(GJs), and basal ectoplasmic specialization (BES) collectively constitute the BTB, and motor proteins localized at BES structures further contribute to the regulation of BTB integrity (11) (Figure 3 illustrates molecular architecture of the BTB between adjacent SCs). The coordinated breakdown, remodeling, and stabilization of the BTB are essential for the orderly progression of spermatogenesis.The mammalian target of rapamycin complex (mTORC) is a Ser/Thr protein kinase complex that regulates cellular status in mammalian cells (12, 13). In the testis, it serves as a key signaling regulator of BTB integrity during the transit of spermatocytes across the barrier (14). mTORC forms two complexes, mTORC1 and mTORC2, with distinct structures and opposing functions (15). mTORC1, assembled in response to stimulatory cues, signals via its downstream target ribosomal protein S6 (rpS6), and enhanced phosphorylation of rpS6 at S235/S236 and S240/S244 leads to inhibition of phosphorylated AKT kinase activity (7). Within the BTB, BESs are composed of two bundles of actin filament arrays between Sertoli cell (SC) plasma membranes, and actin-regulatory protein Arp3 together with actin-bundling protein Eps8 at BESs are indispensable for BTB restructuring (16). By suppressing AKT activity, mTORC1–rpS6 signaling facilitates Arp3 and Eps8 mediated conversion of actin filaments from tightly bundled to branched networks, which drives BES reorganization and renders the BTB transiently permissive (17). Matrix metalloproteinase-9 (MMP-9) is a proteolytic enzyme that can destroy TJs between SC. When mTORC1 reduces AKT phosphorylation, it consequently promotes the secretion of MMP-9. Proteolytic hydrolysis targets TJ components within the BTB, similarly resulting in a short-lived increase in barrier permeability (18). Similarly, mTORC2 functions as a multiprotein complex that activates protein kinase C (PKC) and the small GTPase Rac1, thereby assembling a signaling complex for downstream regulation. In addition, mTORC2 positively regulates AKT, and once this signaling complex is activated it drives Arp3 polymerization and the generation of new actin branches, leading to reconstruction of the BTB (19–22). By tuning its autophosphorylation, focal adhesion kinase (FAK) alters the adhesive properties of complexes containing proteins such as ZO-1 and occludin, directing the cyclic disassembly and reformation of junctional components. FAK exists in two phosphorylated forms, p-FAK-Y397 and p-FAK-Y407, which are localized to BES and TJs in SCs and jointly help preserve BTB integrity (23). Specifically, p-FAK-Y397 is able to bind Arp3–N-WASP and thereby modulate protein conformation (24). However, whether p-FAK-Y397 is directly linked to mTORC1 signaling remains unresolved and represents a promising avenue for future research. Conversely, p-FAK-Y407 counteracts the action of p-FAK-Y397 and promotes the reassembly and closure of separated actin filaments (Figure 4 provides a detailed schematic illustration of the mechanisms underlying BTB regulation.). Recently, research on extracellular vesicles (EVs) has rapidly expanded, and within the testicular interstitium various testicular cell types secrete EVs that carry miRNAs, proteins, and other signaling molecules, mediating intercellular communication across the BTB and thereby contributing to spermatogenesis and immune regulation (25, 26).

### 2.2 Immunoregulation of testicular cells and the testicular microenvironment

SCs are a key structural component of the BTB. TJs formed between adjacent SCs block contact between circulating immune cells, blood and germ cells, providing the anatomical basis for testicular immune privilege (27, 28). In addition, cytokines secreted by SCs are part of the local immune microenvironment, and transforming growth factor (TGF)-β1 produced by SCs protects germ cells from attack by the immune system (29). Activin A, a member of the TGF-β1 superfamily, is secreted by SCs and acts in an autocrine or paracrine fashion on surface receptors. It suppresses the expression of inflammatory mediators such as IL-1 and IL-6 and limits inflammatory responses (30–32). GAS6 (growth arrest specific 6), another factor produced by SCs, serves as an upstream ligand in the TAM signaling pathway and binds the tyrosine kinase receptors Axl, Tyro3 and MerTK on macrophages to mediate phagocytic clearance of apoptotic germ cells (4, 33). LCs synthesize testosterone in the testicular interstitium. Testosterone acts through androgen receptors to regulate SC function and maintain normal testicular immune activity (28). LCs can also modulate the number and function of immune cells such as macrophages in the testis to suppress unnecessary immune activation (34, 35). When exogenous viruses invade, LCs can release pro-inflammatory mediators including TNF-α and interferon (IFN)-α to initiate immune defense (28, 36). Local immunosuppressive factors also contribute to the unique immune microenvironment of the testis. Indoleamine 2,3-dioxygenase (IDO), an immunosuppressive enzyme present in the testicular microenvironment, degrades tryptophan, a key substrate for T cells, thereby effectively suppressing T cell responses and preventing autoimmunity (37). Germ cells and SCs express Fas ligand (FasL) and programmed death ligand 1 (PD-L1) on their surface, which engage Fas and PD-1 receptors on immune cells to induce immune cell apoptosis, limit immune-cell numbers and prevent autoimmune reactions (38–40).

### 2.3 Immunoregulatory functions of testicular immune cell populations

Testicular macrophages account for about 20% of interstitial cells in the testis and are mainly composed of two subsets, classically activated M1 macrophages and alternatively activated M2 macrophages. These two populations have opposing roles: peritubular M1 macrophages exhibit low levels of CD206, CD64, CSF1R and MerTK but high levels of MHCII. They generate NO and reactive oxygen species and express pro-inflammatory cytokines such as IL-6, TNF-α and IL-12. By contrast, interstitial M2 macrophages express lower levels of MHCII and secrete high levels of anti-inflammatory mediators such as IL-10 and TGF-β and they play important roles in anti-inflammatory responses and tissue repair (41). Macrophage polarization is highly plastic. Under different stimuli macrophages can dynamically switch between pro-inflammatory and anti-inflammatory phenotypes, providing potential diagnostic and therapeutic targets for treating autoimmune diseases and controlling inflammation (42). Dendritic cells (DCs), as professional antigen-presenting cells (APCs), primarily function to activate Treg cells. Studies have shown that DCs express IDO, which catalyzes the metabolism of tryptophan into kynurenine. Kynurenine induces the generation of Foxp3^+^ Treg cells and establishes peripheral immune tolerance (43). Under physiological conditions, DCs remain in an immature state and secrete anti-inflammatory cytokines such as TGF-β and IL-10 to suppress excessive activation of effector T cells and further promote the expansion and functional maturation of Foxp3^+^ Treg cells (44). During inflammation, DCs upregulate the maturation markers CD80 and CD86, activate effector T cells and drive inflammatory responses. This process can also contribute to immune infertility (45). Testicular mast cells regulate testicular androgen synthesis and, through secretion of the serine protease tryptase, promote collagen production and fibroblast proliferation (46, 47). Mast cells act as key inflammatory effectors by secreting IL-6 and, via the OX40/OX40L axis, weakening Treg-mediated suppression and promoting Th17 differentiation (48). However, mast cells also exert anti-inflammatory functions. Treg cells can recruit mast cells through IL-9 to limit excessive inflammation and help establish immune tolerance (49). Testicular T cells participate in both cellular and humoral immunity and comprise multiple subsets. Treg cells are closely associated with immune tolerance and prevent excessive autoimmunity by suppressing the activity of other reactive T cells. In humans, Treg cells lack unique lineage-specific markers, but they are generally characterized by high expression of CD25 and Foxp3. Treg cells exert their suppressive functions through several mechanisms, including secretion of inhibitory cytokines, release of granzymes and perforin to induce T cell apoptosis, modulation of metabolic pathways and expression of CD25 to regulate DC function (50). Impaired Treg function or an imbalanced Treg/Th17 ratio has been linked to multiple autoimmune diseases and chronic inflammatory conditions and may be involved in immune-mediated male infertility (51). Th cells, also known as CD4^+^ T cells, arise from naive T cells and comprise several subsets, including Th1, Th2, Th17 and other nonclassical populations. Th1 cells participate in antiviral immunity by producing cytokines such as IFN-γ, IL-2 and TNF-α. Th2 cells secrete IL-4, IL-5 and IL-13 and are involved in antiparasitic immunity and allergic responses. Th17 cells are critical for antifungal defense through the production of inflammatory cytokines IL-17A, IL-17F and IL-22 (52). Tc cells, also known as CD8^+^ T cells, eliminate harmful target cells by releasing FasL, perforin, granzymes and TNF-α. After clearance of target cells, most effector Tc cells undergo apoptosis, whereas a small fraction differentiates into memory cells that support rapid responses upon subsequent antigen exposure (53).

## 3 Epididymal immune microenvironment

The epididymis is a ductal system through which spermatozoa transit upon exiting the testis. Anatomically, it is divided into three primary segments: the caput, corpus, and cauda. Histologically, it consists of a pseudostratified ciliated columnar epithelium supported by an interstitium containing blood and lymphatic vessels (54). Epididymal epithelial cells encompass diverse cell types, including principal, basal, clear cells, and mononuclear macrophages. The blood-epididymal barrier (BEB) is formed by apical TJs between adjacent principal cells. This barrier differs dynamically and molecularly from the BTB maintained by SCs. Through these TJs and selective transport mechanisms, principal cells concentrate specific molecules, such as carnitine and inositol, within the epididymal lumen. This specialized microenvironment is crucial for optimal sperm storage and maturation (55, 56).

Furthermore, distinct regions of the epididymis exhibit markedly different responses to inflammation. Functionally, the caput epididymidis exhibits an incomplete barrier and high permeability. Its primary role is to receive immature spermatozoa bearing novel surface antigens from the testis. Consequently, the caput is relatively insensitive to stimuli and less prone to acute inflammation (57). In contrast, the cauda epididymidis possesses robust immune surveillance capabilities. It completely isolates the lumen from systemic circulation, ensuring mature spermatozoa are stored within an immune-privileged zone to prevent autoimmunity. While this region effectively defends against retrograde urethral pathogens, its intense immune responses can also induce tissue damage (58). Ultimately, this regional immune polarity dictates the specific host defense strategies employed against various diseases and pathogenic factors.

The primary effector cells mediating immunity in the epididymis belong to the mononuclear phagocyte system (MPS), which comprises macrophages (Mφ), DCs and their monocytic precursors (59). The epididymal MPS conducts continuous immune surveillance of the luminal microenvironment without compromising BEB integrity. Basal and intraepithelial Mφ and DCs extend trans-epithelial dendrites through the TJs between adjacent principal cells. This mechanism allows for the real-time detection of luminal spermatozoa and potential pathogens (60).Under physiological conditions, the epididymal MPS is predominantly composed of M2-polarized Mφ and cDC2 cells. These cells collaborate with regulatory T cells to establish an immune-tolerant microenvironment, preventing autoimmune damage to healthy spermatozoa. However, during retrograde pathogen invasion or oxidative stress, this homeostasis is rapidly disrupted as the MPS shifts toward pro-inflammatory phenotypes (M1 and cDC1). The overactivated MPS releases abundant IL-1β, TNF-α, and ROS. Through paracrine signaling, these factors directly mediate the degradation of BEB TJ proteins and induce sperm DNA fragmentation (SDF). Ultimately, this barrier dysfunction triggers the cascade generation of antisperm antibodies (ASA) (61).

## 4 Immune system alterations and therapeutic approaches for male infertility

**Figure 5:**
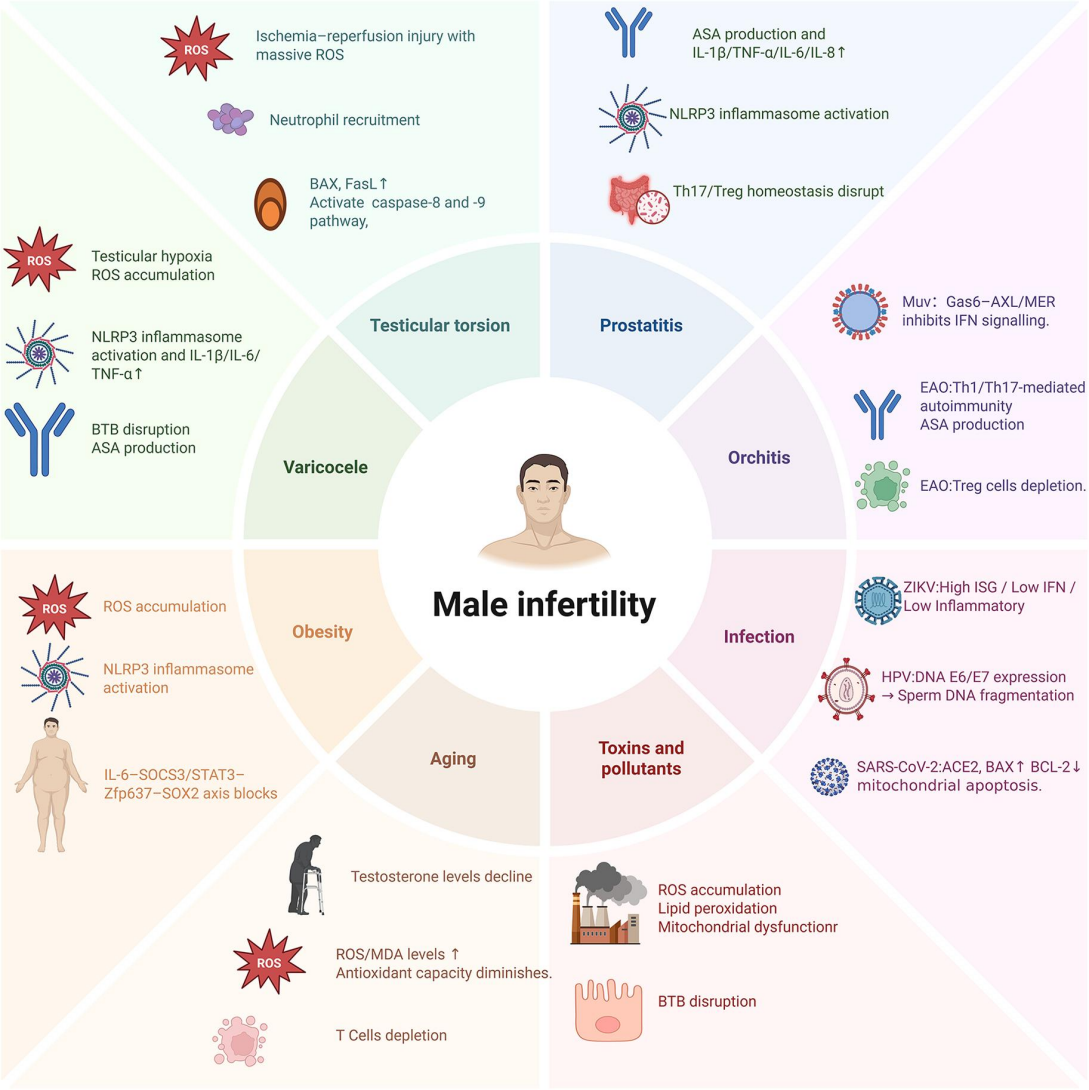
Different diseases leading to male infertility: the underlying mechanisms.

In this review, we use varicocele, testicular torsion, prostatitis, orchitis, viral infection, environmental toxicant exposure, ageing and obesity as representative conditions to explore the immunological mechanisms of male infertility and advances in related therapies, and we summarize the disease-specific mechanisms of male infertility in Figure 5 and Table 1.

**Table 1:** Immunological mechanisms of male infertility.

Disease	Signaling pathway	Key pathological processes
Varicocele	Hypoxia/Venous stasis → ROS → NLRP3 inflammasome → IL-1β/IL-18	Germ cell apoptosis; Mitochondrial dysfunction; Sperm DNA fragmentation (SDF)
	BTB disruption → Sperm antigen leakage → Anti-sperm antibodies (ASA)	Germ cell apoptosis
Testicular Torsion	Ischemic metabolites → Reperfusion-induced ROS accumulation → Release of inflammatory factors	Germ cell injury; DNA structural damage
	Testicular necrosis → Upregulation of BAX and FasL mRNA levels → Activation of Caspase-8 and Caspase-9	Germ cell apoptosis
CP/CPPS	PAMPs → TLR4/NF-κB signaling → Activation of NLRP1 and NLRP3 inflammasomes → Release of inflammatory cytokines	Mitochondrial apoptosis; BTB disruption
	Mast cells → Tryptase/histamine → PAR2	Chronic pelvic pain
Viral Orchitis	MuV → TAM receptors (AXL/MER) → IFN suppression	Sertoli cell lysis;Testosterone suppression
	TLR2/RIG-I → Pyroptosis (GSDMD) → IL-1β	Massive germ cell loss
Autoimmune Orchitis	Treg exhaustion → Th1/Th17activation → IFN-γ/IL-17	Granuloma formation; ASA deposition
Viral Infections	ZIKV → AXL → Interferon stimulated genes(ISG)	Strong ISG;low interferon;low inflammation
	SARS-CoV-2: ACE2 → cytokine storm → Release of inflammatory factors	Germ cell apoptosis;BTB disruption
	HPV:E6/E7 oncoproteins → oxidative stress → DNA damage	HPV DNA integration into the host genome
Environmental Toxins	Xenobiotics (e.g., TCDD/PFOA) → AhR activation → ROS/NF-κB	BTB disruption;oxidative stress
	Heavy metals (Cd/Pb) → mitochondrial uncoupling → ROS	Germ cell apoptosis
Aging	ECM degradation → DAMPs generation → PRR activation → mTORC2 signaling	BTB disruption;inflammatory response activation
	Cellular senescence → SASP → ROS accumulation	Germ cell apoptosis
Obesity	Adipose tissue → leptin → adipose-testis axis → testicular microenvironment cells → systemic inflammation	BTB disruption;oxidative stress

PAMPs, Pathogen-Associated Molecular Patterns; IFN, Interferon; ECM, Extracellular matrix; DAMPS, Damage-associated molecular patterns; PRR, Pattern recognition receptors; SASP, Senescence-associated secretory phenotype.

### 4.1 Varicocele

#### 4.1.1 Immune mechanisms

Varicocele (VC) is a vascular disorder characterized by dilatation of the pampiniform plexus veins and is present in about 40% of men with infertility (62). Traditionally, VC has been considered to impair spermatogenesis and sperm function mainly through local testicular hypoxia and oxidative stress (63), and more recent studies further support an important contribution of inflammation and disruption of the immune microenvironment to varicocele mediated infertility (VMI). Antisperm antibodies (ASA) are widely detected in infertile men, with a prevalence approximately five times higher than in fertile men, and ASA levels decline after surgical correction of VC (64–66). ASA can aggregate sperm and associate with apoptosis related proteins such as caspase 3 and HSP70, thereby inducing apoptosis of sperm cells. In VC patients who are ASA positive, reactive oxygen species (ROS) levels are about 2.8 times higher than in ASA negative VC patients. The study by Bozhemov et al. suggested that VC is an auxiliary factor that increases the risk of ASA production rather than a direct cause of autoimmune disease (64).

Receptor for activated C kinase 1 (RACK1) was first identified as a scaffold protein for protein kinase C, and work by Soares et al. showed that RACK1 regulates phosphorylation of FAK, alters BES, and thereby affects the dynamics of the BTB (67). VC reduces claudin 11 synthesis at TJ, disrupts BTB integrity and causes progressive worsening of barrier damage over time (68, 69). Oxidative stress associated with VC also alters the proportions of testicular immune cells, leading to decreased frequencies of CD8^+^ T cells and macrophages together with increased CD4^+^ T cells, and this immune cell imbalance may contribute to impaired spermatogenesis and the development of an inflammatory seminal microenvironment (70, 71). Inflammatory mediators and their signaling pathways are also important in VC; for example, IL-1α and IL-1β have been implicated in autoinflammatory diseases (72, 73). In VC rat models, testicular expression of IL-1α and IL-1β is markedly upregulated but can be reversed by herbal treatments (74, 75). A case control study found that seminal IL-1α concentrations are higher in men with VC than in fertile controls (36). These findings suggest that IL-1 is one of the major pathogenic mediators contributing to VMI. In men with VC, seminal IL 6 levels are much higher than in fertile men (76, 77). IL-6 promotes nitric oxide (NO) production, aggravates oxidative stress, and reduces sperm motility (78). A recent study by Moretti et al. demonstrated a positive correlation between IL-6 levels and malondialdehyde (MDA) concentrations. Seminal MDA levels correlate negatively with sperm parameters, further supporting a role for IL-6 in inducing sperm damage (79). IL-1 and IL-6 also inhibit the acrosome reaction, thereby impairing sperm function in men. Multiple studies have reported increased TNF-α concentrations in men and animal models with VC (75, 80, 81).TNF-α alters mitochondrial function, enhances NO production, promotes oxidative stress, and suppresses sperm motility, and its role in promoting germ cell apoptosis is under active investigation. Habibi et al.found elevated IFN-γ levels in rat models of VC, indicating that VC can reduce SC numbers and impair sperm quality (82). TGF-β functions as an anti inflammatory mediator that constrains the proliferation of immune precursor cells and maintains a balance between pro inflammatory and anti inflammatory signals (83). IL-1, IL-6, TNF-α and IFN-γ are thus likely to be key immunoregulatory cytokines in patients with VC. At the same time, anti inflammatory mediators such as IL-10, IL-37 and TGF-β are often upregulated to restrain excessive inflammatory responses.

Pattern recognition receptors (PRR) can be divided into five classes, including cytosolic NOD like receptors (NLRs) that are activated by pathogen associated molecular patterns (PAMPs) or danger associated molecular patterns (DAMPs) and ultimately induce transcription of downstream target genes (84). NLRP3, a member of the NLR family, can be directly triggered by cytosolic DAMPs such as peptides, DNA and RNA (85). The adaptor protein-apoptosis associated- speck like protein containing a caspase recruitment domain (ASC) binds NLRP3, promotes cleavage of pro caspase-1, and assembles an active inflammasome complex in macrophages that generates mature IL-1β. In VC rat models, partial ligation of the left renal vein markedly increases NLRP3 gene expression (86), and resveratrol (3,5,4′-triethoxy-trans-stilbene), an anti inflammatory and anti apoptotic compound, can reverse this change through the same pathway (87). By integrating oxidative stress with pro inflammatory cytokine production, the NLRP3 inflammasome represents a promising therapeutic target for future studies. In VC animal models, expression of prokineticin 2 (PK2) is increased, and in models of bacterial orchitis PK2 promotes IL-1β secretion and exacerbates testicular inflammation through the NLRP3 pathway. However, direct evidence for a PK2-NLRP3-IL-1β axis in VC is still lacking and this regulatory module warrants further investigation (88, 89).

The epididymis is a critical site for sperm maturation and plays a key role in regulating sperm motility. Neutral α-glucosidase (NAG), synthesized and secreted by epididymal epithelial cells, serves as a specific marker of epididymal function. In patients with varicocele, seminal plasma NAG levels are significantly reduced and inversely correlated with the severity of the condition (90). Varicocele disrupts epididymal haemodynamics, leading to tissue hypoxia and functional impairment. This dysfunction compromises sperm DNA integrity and motility, ultimately contributing to immune-mediated infertility (91).

In varicocele, venous congestion induces local ischaemia and hypoxia, leading to the accumulation of ROS. This oxidative stress triggers the activation of the NLRP3 inflammasome, promoting the secretion of pro-inflammatory cytokines. These mediators compromise the integrity of the BTB. Consequently, sequestered sperm antigens become exposed to the immune system, eliciting the production of ASAs. Furthermore, these pathological changes impair epididymal function, reducing both sperm motility and DNA integrity.

#### 4.1.2 Therapeutic approaches

**Figure 6:**
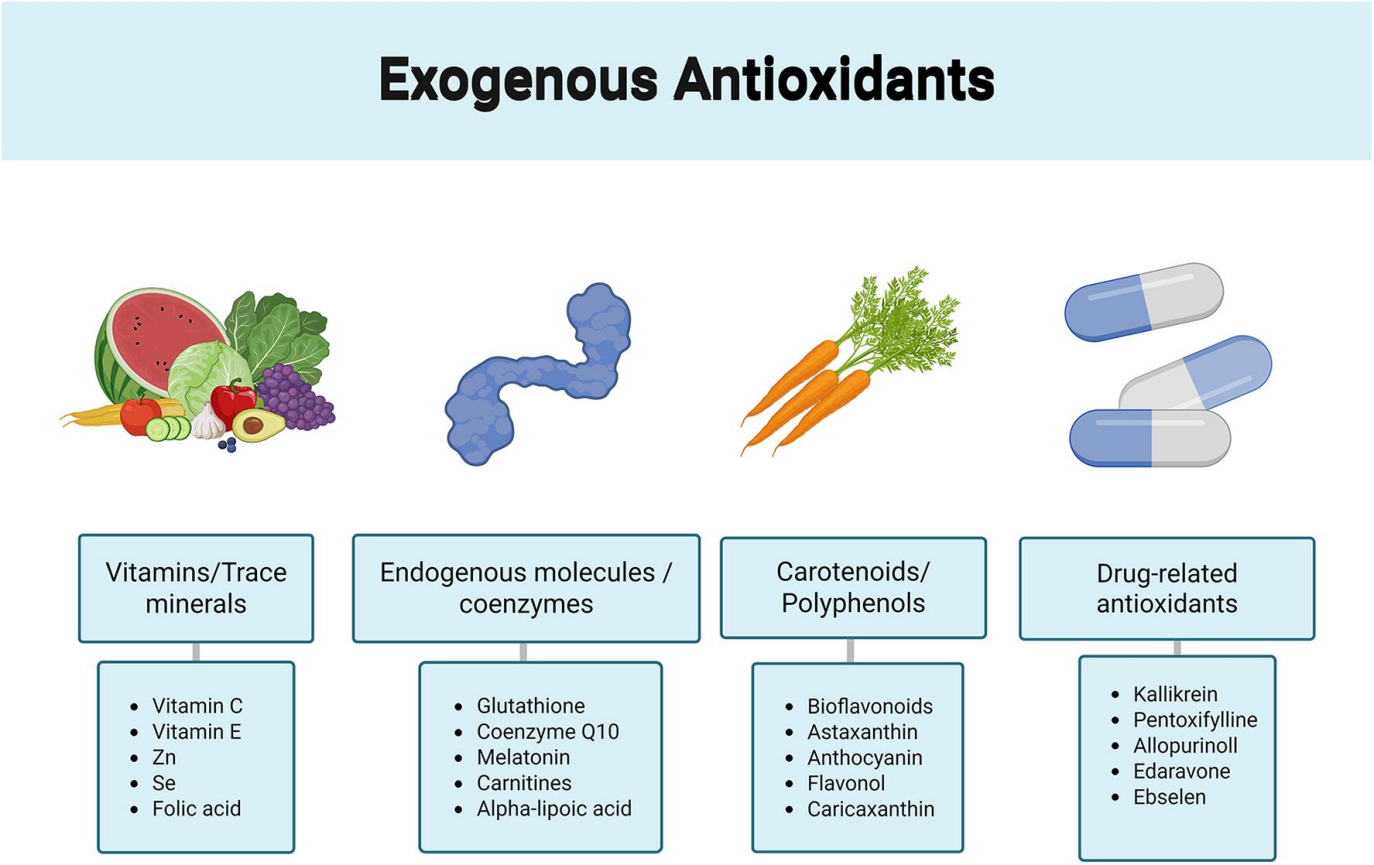
Types of antioxidants.

At present, the first-line therapy for VC remains microsurgical or laparoscopic varicocelectomy, but several studies have shown that ASA positivity attenuates postoperative improvement in semen quality and that a substantial proportion of patients remain infertile after surgery. This persistence may be attributed to sustained immune memory or irreversible damage to the BTB (66). We therefore aim to identify immunomodulatory strategies that can restore spermatogenesis and improve recovery of fertility after surgery. ROS production is central mechanism in VMI. Antioxidant treatment can remove free radicals, preserve sperm DNA integrity and improve mitochondrial function (92). Commonly used antioxidants include vitamin C, vitamin E, zinc, folic acid, coenzyme Q10, astaxanthin, glutathione, selenium, kallikrein, melatonin, pentoxifylline, carnitines and bioflavonoids (Figure 6 illustrates the various types of antioxidants and their classifications) (93). Meta analyses indicate that antioxidant supplementation (AOX) improves sperm concentration and motility in infertile men, and work by Wang et al. further showed that vitamin E increases total sperm count (94, 95). However, excessive intake of antioxidants may cause reductive stress, gastrointestinal symptoms and halitosis. Current guidelines do not provide clear recommendations regarding this issue. Future work should aim to refine recommendations for the rational use of antioxidants. In animal studies, pharmacological interventions for VC have revealed several potential immunometabolic targets. Ghrelin can enhance the activities of antioxidant enzymes such as superoxide dismutase (SOD) and total antioxidant capacity (TAC) in rat models of VC, reduce MDA levels, exert antioxidant and anti inflammatory effects, and decrease inflammatory cytokine production by upregulating PPARγ and downregulating NF-κB (96). In the study by Sara and coworkers, celecoxib (CEB) protected rat testes against atrophy and improved outcomes by increasing TAC and SOD and lowering MDA levels in testicular tissue (96). Dexamethasone (DEX) also attenuates testicular atrophy in rats and reduces the number of atrophic interstitial cells (97). Chlorogenic acid (CGA) is a natural compound with strong anti inflammatory and antioxidant properties, and work by Jia et al. demonstrated that CGA alleviates VMI by reducing mitochondrial damage, inhibiting activation of the NLRP3 inflammasome and increasing ZO-1 expression (98). In work by Lv that combined network pharmacology with animal experiments, the traditional Chinese medicine Mailuoshutong pill (MLST) inhibited the PI3 K/Akt/mTOR pathway and attenuated VC induced oxidative stress and germ cell apoptosis (99). Majid reported that minocycline lowers serum MDA levels and ameliorates VC induced testicular injury through its antioxidant activity (100). A range of agents targeting oxidative stress, inflammation and immune pathways have shown protective effects in VC animal models, but most remain at the preclinical stage and lack high quality clinical trials to establish safety and efficacy; furthermore, not all patients with VC should routinely receive antioxidants (94) Future clinical management should incorporate routine screening for seminal plasma inflammatory markers, such as IL-6 and ASAs. For patients exhibiting elevated inflammatory profiles, surgical intervention combined with anti-inflammatory therapy may offer superior clinical outcomes compared to surgery alone.

### 4.2 Testicular torsion

#### 4.2.1 Immune mechanisms

Testicular torsion(TT) is a frequent urological emergency in children and adolescents that arises from anatomical anomalies of the testis or trauma. When it is diagnosed and surgically treated within 6 h, ischemia–reperfusion injury to the testis can be avoided (101), whereas ischemia lasting longer than 24 h commonly results in irreversible testicular damage, including edema, hemorrhage and germ cell necrosis (102). The mechanisms underlying testicular torsion injury mainly include neutrophil recruitment, generation of ROS, ischemia–reperfusion, lipid peroxidation, microvascular flow alterations and apoptosis (103). When testicular torsion progresses to ischemic necrosis, cells become hypoxic and ATP is depleted so that large amounts of degradation products (xanthine and hypoxanthine) accumulate intracellularly. During the reperfusion phase, a surge of oxygen entry together with xanthine oxidase activity produces large quantities of ROS that damage DNA structure, cause germ cell injury and ultimately lead to male infertility (104). Apoptosis plays a pivotal role in sperm development, and under conditions of testicular torsion, apoptotic pathways may be abnormally activated. Some studies have shown that mRNA levels of pro-apoptotic molecules such as BAX and FasL are increased, and that activation of caspase-8 and caspase-9 pathways enhances germ cell-specific apoptosis (105, 106). Accumulation of inflammatory mediators also contributes to testicular damage. Tamer reported increased expression of TNF-α and IL-6 together with reduced IL-10 after TT, and Turner et al. observed that upregulation of TNF-α, IL-6 and IL-1β further aggravated the inflammatory response (107, 108). During testicular ischemia and necrosis, the BTB is disrupted, sperm antigens become exposed, and ASA are generated that attack germ cells in the contralateral testis and impair normal testicular function (109). In the study by Rodriguez et al. evaluation of contralateral testes in rats with unilateral TT at 30 days after torsion revealed focal damage to seminiferous tubules, significant increases in macrophages, T cells and mast cells in the interstitium, and elevated TNF-α levels, together with detectable ASA in serum (110). In a study by Merve et al., testicular torsion in mice altered macrophage M1/M2 polarization, with an increase in M1 macrophages, and similar changes were found in the contralateral testis (111). However, there is considerable controversy because human data are highly variable (112, 113). One study of 58 patients detected no autoantibodies in the contralateral testis (114), whereas another report found ASA in 8 of 9 patients with TT (115). More recent studies suggest that TT triggers an autoimmune response by disrupting the BTB, and that ASA production may occur regardless of whether the affected testis is removed (102). The central pathophysiology of TT lies in the excessive generation of ROS following ischaemia-reperfusion. This oxidative surge not only causes direct DNA damage and exacerbates spermatogenic cell apoptosis but also triggers the release of inflammatory mediators. These mediators disrupt the BTB, leading to the production of ASAs. Although clinical data on contralateral injury exhibit heterogeneity—likely due to variations in torsion duration and the extent of BTB compromise—the resultant loss of immune tolerance allows ASAs to target testicular tissue, ultimately diminishing male fertility.

#### 4.2.2 Therapeutic approaches

Acute scrotal pain and associated symptoms such as nausea and vomiting caused by TT are relieved by orchiectomy or orchiopexy. However, some studies have indicated that, even after successful surgical intervention, the incidence of testicular atrophy and infertility remains as high as 40%–60% (116), and there is an urgent need to develop novel immunotherapeutic strategies to counter oxidative stress and ischemia–reperfusion injury. Adipose mesenchymal stromal cell-derived exosomes (ADSC-Exos) have shown marked efficacy in ameliorating oxidative stress caused by ischemia–reperfusion injury. ADSC-Exos markedly decrease MDA levels and increase SOD levels after testicular torsion injury, leading to improved sperm quality. In addition, expression of Ki67 in germ cells is increased, which suppresses apoptosis and promotes proliferation of germ cells after testicular injury, and these effects are closely related to activation of the PI3K/AKT and MAPK/ERK1/2 signaling pathways (117). Curcumin is a natural polyphenolic compound widely used to treat inflammatory diseases, and it can inhibit apoptosis induced by hypoxia–reperfusion injury by modulating NLRP3 expression and activation of downstream inflammatory caspases. Formulation with nanoparticles in humans may further enhance its therapeutic potential and bioavailability (118, 119). Rapamycin is a macrolide antibiotic and a specific inhibitor of mTORC1. It can exert antioxidant effects by inhibiting mTORC1 signaling. although it can rescue cells from apoptosis after ischemia–reperfusion injury, it may also induce apoptosis in normal testicular tissue, so the timing and dosage of administration must be carefully evaluated before clinical use (120). Phoenixin-14 (PNX-14) is a newly identified bioactive peptide composed of a 14-amino acid fragment, and it prevents oxidative stress by reducing ROS generation and increasing glutathione levels. It has free radical-scavenging properties and alleviates inflammation by lowering TNF-α and IL-6 levels (121). Nicotinamide mononucleotide (NMN), a precursor of NAD, participates in multiple immune processes and tissue repair. It can increase NAD levels in macrophages and neutrophils By decreasing IL-1β and TNF-α levels and suppressing p-STAT3 and p-p65 expression, it inhibits M1 polarization while promoting M2 polarization (122). During the ischemic and hypoxic phase of TT, endoplasmic reticulum(ER) stress and abnormal protein folding promote apoptosis. Treatment with CGA markedly reduces ER stress markers such as GRP78, IRE1, PERK and ATF6, thereby attenuating testicular injury (123).

To maximize the preservation of fertility potential, future protocols must integrate surgical intervention with perioperative immunomodulation designed to mitigate reperfusion injury and inhibit the inflammatory cascade.

### 4.3 Prostatitis and ascending epididymo-orchitis

#### 4.3.1 Immune mechanisms

Prostatitis is the most prevalent prostate disorder in men, and according to the NIH classification it is divided into acute bacterial prostatitis (type I, ABP), chronic bacterial prostatitis (type II, CBP), chronic prostatitis/chronic pelvic pain syndrome (type III, CP/CPPS) and asymptomatic prostatitis (type IV), with type III accounting for more than 90% of cases (124). Types I and II prostatitis are predominantly caused by bacterial infection. Bacterial invasion of prostatic epithelial and stromal cells induces inflammatory cytokines these inflammatory mediators and metabolites enter the prostatic fluid.They propagate retrogradely via the vas deferens to the epididymis and testis, compromising sperm membrane stability and damaging normal spermatozoa (125, 126).

The precise aetiology of CP/CPPS remains elusive. The epididymis serves as a critical site for the preservation of sperm DNA integrity and the acquisition of motility. Levels of the inflammatory cytokines IL-1β, TNF-α, IL-6 and IL-8 in the seminal plasma are persistently elevated (127). Pro-inflammatory mediators such as IL-1β and TNF-α can markedly induce sperm DNA fragmentation by activating caspase-8 and mitochondrial apoptotic pathways (128), and IL-6 impairs sperm mitochondrial function by inducing inducible nitric oxide synthase (iNOS), reducing ATP synthesis and thereby compromising the energy supply for sperm motility (129). Abnormally elevated levels of chemokines including CCL2, CCL3 and CXCL2 drive the accumulation of inflammatory cells and aggravate the inflammatory response (130). Stimulation by PAMPs or DAMPs triggers persistent TLR4/NF-κB signaling and cytokine upregulation, which compromises BTB integrity and induces the premature release of immature sperm (131). In addition to soluble inflammatory mediators, activation of inflammasomes also plays an important role. Upregulation of NLRP1 and NLRP3 inflammasome components together with increased caspase-1 expression promotes the processing of pro-IL-1β and pro-IL-18 into their mature forms, thereby facilitating release of active inflammatory cytokines and participation in immune responses (132). The precise etiology of CP/CPPS has not yet been fully elucidated, but autoimmunity is considered a major contributor. In a model of autoimmune prostatitis induced in non-obese diabetic (NOD) mice, prostate steroid-binding protein (PSBP) was identified as an autoantigen recognized by the NOD immune system (133). In a study by Motrich et al., deficiency of Th1 cytokines in experimental autoimmune prostatitis (EAP) mice reduced susceptibility to EAP, whereas Th1 and Th17 cells, through adaptive immune responses mediated by secretion of IFN-γ, IL-17A and related cytokines, were shown to be closely associated with chronic prostatic inflammation (134). Xu et al. reported that trypsin released from degranulating mast cells activates protease-activated receptor 2, thereby aggravating neurogenic inflammation. This mechanism drives a pathological loop encompassing chronic pain, endocrine dysfunction, and reproductive impairment (135).Moreover, dysbiosis of the gut microbiota may disrupt the balance between Th17 and Treg cells, and a reduction in the microbial metabolite propionate can aggravate autoimmune inflammation in the prostate (136). The interplay of autoimmunity, neuroimmunity, and gut dysbiosis, alongside an inflammatory seminal microenvironment, synergistically drives the pathogenesis of infertility in CP/CPPS patients.

#### 4.3.2 Therapeutic approaches

In patients with ABP, systemic signs of infection are common, and the main therapeutic strategy consists of adequate courses of antibiotics in combination with analgesia, fluid replacement and supportive measures such as abscess incision and drainage or urinary catheterization when necessary (137). For CBP, prolonged antibiotic therapy remains the cornerstone of treatment and is often combined with agents targeting lower urinary tract symptoms to achieve better therapeutic outcomes (138). The pathogenesis of CP/CPPS remains unclear and may involve autoimmunity, which makes it challenging to deliver causal treatment. Current therapeutic strategies therefore focus mainly on pain control, anti-inflammatory treatment and management of psychological factors. Release of mast cell mediators is a key driver of pain. In patients with CP/CPPS, expression of tryptase-β and the protease-activated receptor PAR2 is increased, which regulates phosphorylation and signaling of the downstream kinase ERK1/2 and contributes to the development of pain (130, 139). Mast-cell-related mechanisms therefore provide a novel avenue for treating pain in CP/CPPS. The development of CP/CPPS may also be related to infection with multiple bacterial species, and bacteria can evade immune attack and reduce antibiotic efficacy by forming biofilms. A growing number of studies have explored targeting distinct signaling pathways in the treatment of CP/CPPS. Lycium barbarum polysaccharide (LBP), which has multiple biological activities including immunomodulatory and hypoglycemic effects, can attenuate systemic inflammation by inhibiting Th17 cell differentiation through TRAF6 suppression of the JAK–STAT pathway (140). Dietary intake of docosahexaenoic acid (DHA) can reduce the severity of EAP by inhibiting Th17 cell development through the PPARγ/NF-κB/IL-17A pathway (141). Sulforaphane (SFN) is an isothiocyanate abundant in cruciferous vegetables. SFN alleviates inflammation and mitigates EAP symptoms by activating the transcription factor Nrf2 to maintain cellular homeostasis and by suppressing the NLRP3 inflammasome via the Nrf2/HO-1 axis (142). Xialiqi capsule, a commonly used preparation for treating benign prostatic hyperplasia, may also have therapeutic potential in CP/CPPS. It regulates apoptosis of prostatic epithelial cells and suppresses the NLRP3/caspase-1 pathway, leading to decreased release of inflammatory cytokines (143). In clinical practice, therapeutic strategies for CP/CPPS should prioritize restoring the immune microenvironment and metabolic homeostasis of seminal plasma.

### 4.4 Orchitis

#### 4.4.1 Immune mechanisms

Clinically, orchitis impairs sperm production and can lead to male infertility. Mumps is a contagious disease caused by mumps virus (MuV), mainly transmitted via respiratory droplets, and it can result in male infertility (144). Experimental studies have elucidated the mechanisms of MuV-mediated infertility in mice, showing that sialic acids (SA) on the surface of SCs and LCs mediate MuV entry into testicular cells (145). AXL and MER, members of the receptor tyrosine kinase TAM subfamily, interact with their ligands Gas6 and protein S, which bind to the viral envelope. The receptor ligand complex modulates testicular immune responses, suppresses antiviral IFN signaling and facilitates MuV replication (146). MuV activates TLR2 and cytosolic RNA sensor MDA5/RIG-I signaling pathways, thereby inducing the expression of multiple immunoregulatory cytokines, including the inflammatory mediators TNF-α and IL-6, the chemokines CXCL10 and MCP-1, and type I interferons IFN-α and IFN-β (147, 148). CXCL10 produced by SCs in response to MuV stimulation can induce germ cell apoptosis, while TNF-α disrupts the integrity and permeability of BTB (149). MCP-1 and CXCL10 produced by SCs, LCs and TMs are likely to recruit leukocytes (L), exacerbating local inflammation and tissue damage (150). Recently, work using *ex vivo* human testis tissue has shown that MuV preferentially infects LCs, SCs and TMs, eliciting a pro-inflammatory response largely driven by IL-1β and IL-18. Release of IL-1β and IL-18 appears to be tightly associated with GSDMD-mediated pyroptosis. MuV infection of the human testis can also suppress LC testosterone production by impairing the 17,20-lyase activity of CYP17A1 (151). Autoimmune orchitis (EAO) is distinct from other types of orchitis, being defined by concurrent ASA production and testicular inflammation. It results from immune attack against testis specific components. Autoimmune disease can be triggered by diverse factors, and once initiated the testicular immune barrier is disrupted, sperm antigens become exposed and antigen–antibody reactions are elicited (152). T cells constitute a central arm of the immune system. During EAO CD4^+^ T cells, especially Th1 and Th17 populations, are activated and enriched in the testis, where they secrete IFN-γ, IL-1, IL-6 and other inflammatory cytokines that both directly injure the seminiferous epithelium and drive B cells to produce ASA, thus worsening tissue damage and dysfunction (153). ASA may further engage Fc receptors on interstitial MPs, resulting in antibody deposition and amplification of local inflammation (154). Tregs are crucial for maintaining immune tolerance, and depletion of Tregs leads to increased infiltration of CD4^+^ T cells, monocytes and MPs together with enhanced ASA production. Sperm bound by ASA are more prone to agglutination and phagocytosis, thereby compromising male fertility (155), and Treg-mediated immune tolerance to sperm is therefore essential for preserving male fertility, with its breakdown representing an important pathological basis of autoimmune male infertility. A pivotal aspect of managing orchitis lies in accurately discerning its immunological etiology: distinguishing between an inflammatory cascade and the breakdown of immune tolerance. This determination dictates the therapeutic approach, as misinterpretation risks treatment failure and potential aggravation of testicular injury.

#### 4.4.2 Therapeutic approaches

Infectious orchitis is largely self-limited, and at present no virus-specific therapeutic regimens are available. Accordingly, clinical care focuses on supportive treatment including bed rest, scrotal support and antipyretic, analgesic and anti-inflammatory medications, and symptoms usually improve within about one week in most patients (156). Vaccination remains the most effective strategy for reducing MuV infection and preventing its complications (157). The use of interferons to treat infectious orchitis has long been controversial. In one earlier study, treatment of mumps orchitis with interferon-α-2β led to seminiferous tubule atrophy in 40% of patients (158), and in another study of 56 patients treated with interferon-α after mumps orchitis, only two were considered free of sequelae (159). At present, there is no evidence to support interferons as first-line therapy for MuV orchitis, and future efforts should focus on developing more precise antiviral strategies based on receptor–ligand interactions or viral replication mechanisms. In experimental EAO models, researchers have achieved therapeutic benefit by depleting macrophages (160), neutralizing inflammatory cytokines (161, 162) or administering testosterone. Testosterone acts as an immunosuppressive factor in testicular inflammation; supplementation of testosterone in rats can inhibit the progression of EAO (163). Gasdermin D (GSDMD) is a pore-forming protein that serves as a key downstream effector of inflammasome-mediated pyroptosis (164). Loss of GSDMD in macrophages reduces inflammatory responses and limits sperm damage (165). It has emerged as a potential therapeutic target. Recent studies have shown that VD3, a biologically active vitamin D metabolite, can shift testicular immune regulation toward a tolerogenic microenvironment by inhibiting T cell accumulation and increasing production of the anti-inflammatory cytokine IL-10, which delays the course of the immune response (166). Building on these findings, future studies that integrate Treg-related pathways with the inflammasome–pyroptosis axis are expected to identify reliable biomarkers and potential therapeutic targets for EAO.

### 4.5 Infection

#### 4.5.1 Immune mechanisms

Zika virus (ZIKV) is an RNA virus that can be transmitted by mosquito vectors and can also spread through sexual contact. When pregnant women are infected with ZIKV, the virus can damage fetal central nervous system cells and lead to microcephaly in newborns (167). The virus can replicate within the human testis, impairing normal testicular function, and it is able to infect SCs, LCs, TMs and germ cells (168). Studies have shown that although germ cells and SCs are infected by ZIKV, disruption of the BTB has not been observed. Infiltration of macrophages carrying virus across the BTB into the seminiferous tubules may represent a novel route of infection (149, 169). In testicular explants, ZIKV induces the expression of multiple antiviral genes, including interferon-stimulated genes (ISGs) such as ISG15 and RSAD2, but it does not increase secretion of type I, II or III interferons by the explants (170). Moreover, ZIKV infection of human testes induces little or no increase in pro-inflammatory cytokines apart from CXCL10, resulting in a unique “strong ISG/low interferon/low inflammation” immune profile that favors persistent viral presence in the testis and in sperm (171).

Human papillomavirus (HPV) infection is the most common sexually transmitted disease worldwide and is mainly transmitted through sexual activity and skin or mucosal contact (172). While HPV infection in males is predominantly asymptomatic, the virus is ubiquitous throughout the reproductive tract, having been detected in the urethra, vas deferens, testes, epididymis, and prostate (173). HPV infection is one of the causes of male infertility and may be associated with SDF in the testicles and epididymis. SDF is a form of sperm cell damage and is strongly associated with reduced embryo quality and increased miscarriage rates in women (174, 175). Connelly et al. reported that DNA fragments driven by the E6–E7 regions of HPV16 or HPV31 increase SDF and promote apoptosis of sperm cells (176). Recent studies have shown that the DNA fragmentation index (DFI) is markedly elevated in men with HPV infection, with the risk of increased DFI exceeding 30% in some cohorts (177). Oxidative stress may be an important intermediate mechanism by which HPV induces sperm DNA damage. In HPV-positive men, levels of reactive oxygen species (ROS) in seminal plasma are elevated, and superoxide dismutase (SOD) activity is also increased, which is considered a compensatory response to oxidative stress (178). In the future, monitoring SDF may help to evaluate the effects of different antioxidants and provide a new direction for the treatment of HPV-associated infertility (179, 180). Besides, more than 40% of HPV-infected men have ASA bound to the surface of their sperm, whereas ASA levels are low in men without HPV infection (181).

Severe acute respiratory syndrome coronavirus 2 (SARS-CoV-2) is an RNA virus, and coronavirus disease 2019 (COVID-19), first identified in 2019, is caused by this coronavirus (182). COVID-19 has affected the health of more than 500 million people, and beyond its impact on the respiratory tract it may exert long-term effects on male reproductive health (183). Angiotensin I-converting enzyme 2 (ACE2) receptors are expressed on LCs and SCs in the testis, and COVID-19 can enter the testis by binding to ACE2 and thereby damage testicular tissue (184). Testicular biopsy specimens from affected patients have shown damage to seminiferous tubules, reduced numbers of LCs with accompanying inflammation, and even visible viral particles on electron microscopy (185). COVID-19 can also stimulate production of inflammatory cytokines such as IL-6, IL-8, IL-10 and TNF-α, while testosterone and inhibin B levels decline. Infiltration of these cytokines disrupts BTB integrity and reduces the expression of junctional proteins including occludin, claudin-11 and ZO-1 (186, 187). Moreover, after SARS-CoV-2 infects testicular cells, expression of ACE2, BAX and caspase-3 is increased, whereas the anti-apoptotic protein BCL2 is downregulated, leading to mitochondrial dysfunction, activation of apoptotic pathways, sperm membrane lipid peroxidation, DNA damage and apoptosis, all of which compromise fertility (188, 189).

In addition to viral infections, bacterial pathogens such as neisseria gonorrhoeae, mycoplasma, and uropathogenic escherichia coli are prominent causes of epididymitis associated with male infertility. The ascending extension of inflammation from the epididymis to the testis, disrupts the local microenvironment, thereby impairing sperm maturation and motility (190). Chlamydia infection typically manifests as urethritis, chronic or untreated infections may induce fibrotic remodeling and urethral strictures, resulting in physical obstruction of the reproductive tract (191). Immunologically, the recognition of these pathogens by host immune cells triggers a robust inflammatory response, characterized by the upregulation of pro-inflammatory cytokines, including IL-6, IL-8, and TNF-α. This cytokine surge exacerbates oxidative stress and the accumulation of ROS, which compromises sperm membrane integrity and may suppress LC steroidogenesis, leading to reduced testosterone levels (131).

#### 4.5.2 Therapeutic approaches

For bacterial and viral infections, prevention of exposure and vaccination remain the primary strategies. After infection has occurred, emerging effective therapeutic strategies for these diseases continue to be explored. Antibiotic therapy remains the cornerstone of the clinical management of bacterial infections. Current evidence and clinical guidelines identify ceftriaxone and doxycycline as the first-line therapeutic regimens for neisseria gonorrhoeae and chlamydia or mycoplasma infections. However, the escalating threat of antimicrobial resistance compromises therapeutic efficacy, underscoring the imperative for vigilant surveillance and strict antibiotic stewardship (192). In ZIKV-infected germ cells, type I interferons and antiviral effector molecules are lacking, and these cells are protected by the BTB from adaptive immune responses, which together promote persistent viral infection. Studies have shown that exogenous IFN-β enhances the antiviral immune functions of testicular germ cells and inhibits ZIKV replication in *ex vivo* testes (193). In the study by Wang et al., a model of ZIKV infection was established and C-type lectin domain family 5 member A (CLEC5A) was identified for the first time as a factor that exacerbates ZIKV infectivity. CLEC5A is a C-type lectin-like receptor, and in mice with normal CLEC5A expression, excessive activation of inflammatory cytokines enhances leukocyte transmigration across the BTB, resulting in testicular tissue damage and reduced sperm motility. Conversely, this inflammatory response is attenuated in CLEC5A deficient mice, indicating that the CLEC5A/DAP12 axis is a promising target for alleviating ZIKV-induced orchitis (194). IFN-ε is expressed in LCs and TMs and can be detected in the testicular interstitium. By upregulating ISGs and downregulating pro-inflammatory cytokines, IFNε markedly suppresses ZIKV replication in human SCs and thus represents a novel intervention target for ZIKV-associated male infertility (195). For infertile men with HPV infection, two emerging strategies are currently available: sperm washing and adjuvant HPV vaccination. Treatment of HPV-positive sperm with heparinase III can completely eliminate HPV DNA, but this enzyme has not yet been widely approved for use in assisted reproduction (196). Implementation of a hyaluronidase-based sperm washing (IALu) protocol, which disrupts the syndecan-like glycosaminoglycan components that mediate HPV attachment to the sperm surface, can eliminate HPV adherent to sperm. This procedure has already been adopted in some assisted reproduction centers (197). HPV vaccination not only prevents new infections but also improves the prognosis of previously infected patients. After vaccination, the time required to clear HPV is significantly shortened, viral virulence is attenuated and the risk of recurrence is reduced, which may aid in the management of male infertility (198). For SARS-CoV-2, no specific therapeutic regimen is currently available and most studies remain at the basic research stage, so avoiding infection or reinfection as far as possible remains the best “treatment” strategy.

### 4.6 Toxins and environmental pollutants

#### 4.6.1 Immune mechanisms

Behind rapidly developing modern cities, various environmental pollutants and occupational exposures are increasingly becoming risk factors for male reproductive health. Many toxicants can induce oxidative stress and inflammatory responses, disrupt the testicular immune microenvironment and the BTB, and ultimately reduce male fertility. 2,3,7,8-Tetrachlorodibenzo-p-dioxin (TCDD) is an organic pollutant that mainly originates from waste incineration, metal smelting, manufacture of pesticides, herbicides and bleaching agents (199). TCDD can trigger TNF-α and MAPK pathways, leading to inflammation, oxidative stress and apoptosis of germ cells. At the same time it inhibits VEGF signaling, disrupting testicular angiogenesis (200). Microplastics (MPs, <5 mm) and nanoplastics (NPs, <1 µm) are accumulating in oceans, rivers and soils as global plastic production increases, and they enter the food chain, threatening human health (201). MPs and NPs damage male reproductive function by reducing the expression of tight junction proteins such as claudin-11, occludin and ZO-1, thus breaking down the BTB in mice. They trigger lipid peroxidation and mitochondrial dysfunction, decrease sperm number and motility, and impair the steroidogenic enzymes P450scc, P450c17, 3β-HSD and 17β-HSD, thereby disturbing testosterone production in LCs (202). Perfluorooctanoic acid (PFOA) and perfluorooctane sulfonic acid (PFOS) are highly fluorinated aliphatic compounds widely used in food packaging, lubricants and firefighting foams. Because of their high stability and long half-lives, they accumulate extensively in the environment and in living organisms (203). However, these compounds have multiple toxic effects on male reproductive health. PFOA can induce oxidative stress, activate apoptotic pathways and thereby promote germ-cell death and autophagy. PFOA further impairs the BTB by engaging the TNFα/p38 MAPK signaling axis. At the same time, it interferes with several signaling proteins and pathways, such as p-FAK-Y407 and the mTORC1/rpS6/Akt1/2 cascade, inducing cytoskeletal degradation and thereby disrupting the BTB architecture (204, 205). Cisplatin is a chemotherapeutic agent widely used to treat various malignancies, but it exhibits marked reproductive toxicity. Heavy metals such as cadmium, lead and mercury are long-standing public health concerns and have repeatedly been implicated in cases of male infertility worldwide. Exposure to heavy metals can occur through contaminated food and drinking water, polluted soil and cigarette smoke, leading to intoxication. Heavy metals can uncouple mitochondrial oxidative phosphorylation and induce intense oxidative stress, apoptosis and autophagic imbalance in the testis and other organs, ultimately leading to male infertility (206). Food-related contaminants have also attracted worldwide attention in recent years. Aflatoxins (AFTs) are important mycotoxins and are regarded as major contributors to environmental and food contamination, among which aflatoxin B1 (AFB1) is considered the most toxic compound, and aflatoxins can disrupt BTB function by altering the expression of several key tight junction proteins. AFTs also induce ROS production and lipid peroxidation, activate the NF-κB pathway, suppress the Nrf2 antioxidant axis, enhance the expression of inflammatory cytokines such as IL-1β and TNF-α, exacerbate inflammation and impair spermatogenesis (207, 208).

#### 4.6.2 Therapeutic approaches

For male infertility caused by toxicants and environmental pollutants, primary prevention by minimizing exposure remains the most effective and economical “treatment.” Increasing numbers of studies are exploring antioxidant and immunomodulatory approaches to mitigate testicular injury caused by these toxicants. Gum Arabic/Acacia senegal (GA) is a natural compound with antioxidant properties. In a study by Ayesha et al., zinc sulfate, L-carnitine, lycopene and coenzyme Q10 were shown to improve sperm count and motility by lowering ROS levels, and to counteract toxicant exposure by rebalancing pro-apoptotic and anti-apoptotic genes, thereby treating toxin-related male infertility (209). Several clinical studies have confirmed that small molecules (such as caffeic acid and rapamycin), probiotic supplements (ZG7, Lactobacillus casei and Bacillus subtilis) and camel milk can activate the Nrf2-ARE antioxidant pathway and inhibit NF-κB-mediated inflammatory responses (210, 211). Melatonin, acting as an antioxidant and radical scavenger, helps to mitigate the adverse effects of chemotherapeutic agents and to protect testicular tissue from heavy-metal-induced injury (212). The aryl hydrocarbon receptor (AhR) is a ligand-activated transcription factor, and recent studies suggest that it serves as a key signaling hub linking environmental toxicants to male reproduction. AhR activation can induce ROS, whereas AhR antagonists such as resveratrol and curcumin may confer protection. Targeting AhR regulation may represent a novel strategy for preventing and treating toxicant-related male infertility (213).

### 4.7 Aging

#### 4.7.1 Immune mechanisms

Aging is a gradual process, and in men serum testosterone concentrations decline progressively with increasing age, this fall in hormone levels alters sperm quality. Erectile dysfunction (ED) is likewise closely associated with advancing age and reduced testosterone concentrations (214). Recently, the concept of testicular immunosenescence has been proposed, positing that T cell exhaustion, Treg dysfunction, abnormal macrophage polarization and disruption of the BTB form the immunological basis for the decline in fertility (42). Oxidative stress is central to testicular aging (215). Under physiological conditions, ROS promote sperm maturation and capacitation by activating tyrosine phosphorylation and enhancing cell signaling, but in older individuals excessive ROS attack membrane lipids and proteins, and lipid peroxidation products such as MDA accumulate in the testis and damage germ cells and SCs (216). Aging increases the mutation rate of mitochondrial DNA and reduces mitochondrial efficiency, thereby further weakening the antioxidant capacity of germ cells (217, 218). Aging reshapes the testicular immune microenvironment, with higher levels of pro-inflammatory cytokines such as TNF-α and IL-6, and RNA sequencing has shown that the expression of inflammation-related genes is markedly increased in SCs (219). It also alters macrophage phenotypes, increasing the proportion of pro-inflammatory M1 cells and reducing anti-inflammatory M2 cells, so that testicular immune homeostasis gradually shifts toward a pro-inflammatory state (220). Aging can also alter the extracellular matrix (ECM), whose degradation produces collagen and other fragments that act as DAMPs. These molecules can bind PRRs and trigger inflammatory responses. In addition, continuous infiltration of inflammatory cells and progressive weakening of TJs between SCs compromise BTB integrity, and inflammatory cells may further affect the BTB by interfering with the mTORC2 pathway, making it easier for immune cells to damage the reproductive system (221–223).

#### 4.7.2 Therapeutic approaches

For infertility related to testicular aging, current therapeutic strategies mainly focus on four aspects: reducing oxidative stress, suppressing chronic inflammation, protecting mitochondrial function and delaying aging of the immune system. Melatonin is a pineal hormone that lowers testicular ROS and MDA levels, increases antioxidant enzyme activities and modulates androgen production (224, 225). It can also reduce the expression of the NLRP3 inflammasome and pro-inflammatory cytokines such as IL-1β, thereby improving testicular inflammation (226, 227). Icariin, the principal active component of the traditional Chinese herb Epimedium, has a long history of use in treating male aging and hypogonadism. It can upregulate ERα/c-fos-PKR signaling to attenuate TJ damage in SCs, increase the number of SCs, enhance expression of TJ associated proteins (ZO-1 and occludin) and ES related protein β-catenin (228). Declining NAD^+^ levels are an important hallmark of organismal aging and mitochondrial dysfunction. Supplementation with NAD^+^ precursors can slow the decline in mitochondrial function and counter immune aging (229, 230), in part by activating the protective deacetylase SIRT1, improving energy metabolism and enhancing immune function. More studies are needed to define the optimal dosing for interventions that target NAD^+^ in male infertility (231). Preserving mitochondrial function is another way to combat infertility. Elamipretide (SS-31) is a peptide that targets mitochondria, improves mitochondrial function and organelle membrane integrity and reduces oxidative damage. In the reproductive field *in vitro* studies have shown that SS-31 alleviates oxidative damage to male sperm during cryopreservation and improves post-thaw sperm motility, mitochondrial function and DNA integrity. But the long term effects of SS-31 on testicular toxicity have not yet been systematically evaluated and require further investigation (232, 233). At the molecular level, overexpression of miR-143-3p promotes SC senescence and downregulates the expression of TJs. Inhibition of TGF-β receptors can ameliorate age-related BTB damage in mice by downregulating miR-143-3p. It may represent a new strategy to combat male infertility (234). Emerging approaches such as single cell RNA sequencing and spatial transcriptomics are transforming our understanding of testicular aging. It is hoped that in the future precision medicine technologies will enable gene editing to modulate overall immune responses (235, 236).

### 4.8 Obesity

#### 4.8.1 Immune mechanisms

Obesity is a global health problem defined as a body mass index (BMI) ≥ 30 kg/m^2^ and characterized by abnormal accumulation of body fat. An increasing body of evidence indicates that obesity promotes male infertility via chronic low-grade inflammation, abnormal adipokine secretion and imbalance of oxidative stress (237). Obesity induces cells within the testicular microenvironment to secrete pro-inflammatory cytokines such as IL-6 and TNF-α, thereby aggravating inflammation. Elevated IL-6 activates the SOCS3/STAT3 signaling pathway, suppresses zinc finger protein 637 (Zfp637), abnormally upregulates SRY-box transcription factor 2 (SOX2) in spermatogonia, thereby damaging spermatogonia in mice (238). TNF-α activates the transcription factor NF-κB, induces apoptosis of germ cells and disrupts TJs in SCs, ultimately leading to male infertility (239, 240). Obesity-related inflammation further comprises activation of TLR4, endoplasmic reticulum stress, mitochondrial dysfunction and aberrant activation of serine/threonine kinases (241). Furthermore, high fat intake elevates NLRP3 inflammasome levels, breaking immune tolerance and reducing both sperm number and motility (242, 243). Chronic inflammation causes excessive generation of ROS, damages LCs and promotes apoptosis of germ cells (244). Accumulation of ROS also alters the expression of BTB proteins such as ZO-1 and CX43 (245, 246). Abnormal adipokine secretion in obese men impairs testicular function. Obesity-induced hyperleptinemia binds to receptors on seminiferous tubular cells to activate the PI3 K pathway, increasing free-radical production and reducing antioxidant enzyme activity, which leads to oxidative stress and impaired spermatogenesis (247).

#### 4.8.2 Therapeutic approaches

For male infertility caused by obesity, weight loss and lifestyle modification are the most effective measures. Reduction of abdominal fat has been shown to decrease oxidative stress and to improve sperm quality and morphology (248). Improving dietary patterns can likewise ameliorate obesity-related metabolic disturbances and sperm quality. The Mediterranean diet is a representative dietary pattern that includes fruits, vegetables, unsaturated fatty acids, grains, dietary fiber and foods rich in antioxidant components (249–251). Such dietary patterns help reduce systemic inflammation and oxidative stress, improve insulin resistance and metabolic disturbances. These indirectly improve male reproductive parameters. Metabolic modulators and anti-inflammatory drugs can also be used to treat male infertility. Metformin, which is widely used to treat type 2 diabetes and insulin resistance, can increase the numbers of SCs, spermatogonia and LCs in the testis by reducing testicular cell atrophy, lowering oxidative stress and activating the MAPK pathway (252). In obese individuals, antioxidant enzyme activity is reduced. Current studies have shown that antioxidants such as coenzyme Q10 and unsaturated fatty acids can lower DFI, but their impact on the oxidation–reduction potential (sORP) remains limited. Antioxidant therapy alone is unlikely to cure male infertility and should be combined with weight loss and metabolic modulation (253, 254). With advances in bariatric surgery, whether weight loss after sleeve gastrectomy can improve male infertility remains to be determined. Some studies have reported postoperative oligozoospermia and even azoospermia, which may be related to rapid weight loss and excess ROS generated by catabolism. The long-term impact of bariatric surgery on infertility in obese men still requires clarification in large cohort follow-up studies (255). Mesenchymal stem cell-derived exosomes (MSC-Exos) are an emerging therapy for improving testicular function. They can modulate the testicular immune microenvironment, improve sperm parameters and increase sperm adhesiveness by upregulating adhesion molecules. It may provide a novel treatment for obesity-related immune male infertility (256).

## 5 Discussion

The testes and epididymis are complex organs possessing a unique immune microenvironment. To translate mechanistic insights into immunological male infertility into clinical therapies, we must gain an in-depth understanding of how immune cells and immune mediators act within the testicular microenvironment and implement targeted interventions. Unlike previous reviews, which have predominantly concentrated on single aetiologies (e.g., varicocele) or isolated pathological mechanisms (e.g., oxidative stress), this review distinguishes itself by systematically integrating the immunological commonalities across eight distinct conditions—ranging from structural abnormalities to toxic exposure and metabolic disorders.

**Figure 7:**
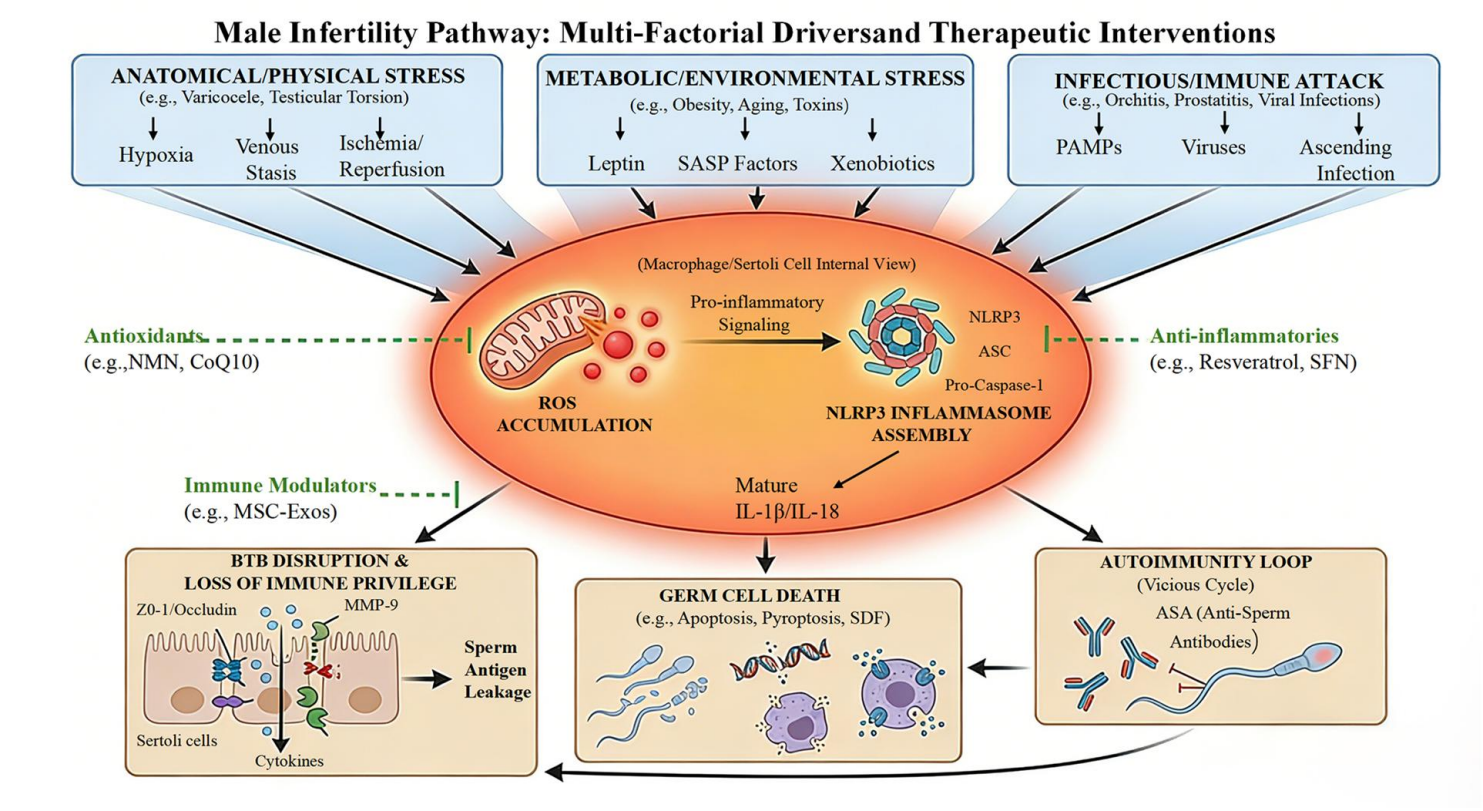
Integrative schematic of immune pathways in male infertility: from diverse etiologies to convergent pathology. This figure illustrates the convergent mechanisms underlying immunological male infertility, which are driven by the core “ROS-NLRP3 inflammasome-cytokine” axis. Diverse upstream stress signals trigger ROS accumulation and inflammasome activation within the central immune hub, precipitating three major downstream pathological consequences: disruption of the BTB, germ cell death, and the production of ASA. Green annotations highlight therapeutic interventions targeting distinct nodes of this axis, such as antioxidants, NLRP3 inhibitors, and immunomodulators.

When reviewing current interventions, antioxidant therapy emerges in nearly all treatment modalities. This therapeutic commonality stems from shared pathogenic pathways. Specifically, chronic inflammation and oxidative stress mutually amplify each other. Furthermore, the disruption of BTB architecture, loss of immune tolerance, and immune imbalance represent common pathological mechanisms across diverse diseases. The core pathogenic cascade comprises ROS overproduction, inflammasome activation, BTB disruption, and spermatogenic cell injury (Figure 7 illustrates the convergent mechanisms underlying immunological male infertility). Consequently, broad-spectrum antioxidants, such as vitamin E and coenzyme Q10, serve as universal therapeutic cornerstones across these conditions.

Although antioxidant agents are proven effective against immune-mediated infertility, they remain a double-edged sword. Meta-analyses confirm the efficacy and safety of oral antioxidants in improving sperm concentration and motility. However, heterogeneous study designs and the multifactorial etiology of infertility obscure the optimal antioxidant regimen (257, 258). Under physiological conditions, ROS are not detrimental to spermatozoa. Instead, moderate ROS levels act as crucial signaling molecules for physiological processes, including sperm capacitation, the acrosome reaction, and oocyte fusion (95). Clinically, administering excessive high-dose antioxidant supplements disrupts the redox homeostasis of seminal plasma, leading to reductive stress. This reductive state induces abnormal sperm chromatin decondensation and disrupts signaling pathways, ultimately impairing male fertility (30259539). Therefore, defining the safe threshold for antioxidant therapy remains an urgent clinical challenge.

Beyond antioxidant therapy, the clinical value of other targeted treatments lies in the precise blockade of specific disease triggers. For example, in environmental toxicant exposure models, agents like resveratrol act as AhR antagonists to abolish targeted receptor activation. For EAO, restoring regulatory Treg cell-mediated immune tolerance is paramount for effective treatment. Additionally, in viral infection models such as Zika, modulating the IFN pathway or specifically blocking receptors can curb the systemic spread of infection. Consequently, future therapeutic paradigms will inevitably shift from broad-spectrum antioxidation toward precise immunomodulation.

Numerous natural products, peptide hormones and targeted agents designed to counter inflammatory responses remain in preclinical animal studies. Future work will require additional clinical trials and careful evaluation of potential human toxicities to prevent adverse reactions. Anti-inflammatory therapies can inhibit key inflammatory signaling pathways, modulate macrophage polarization, enhance T cell function and reshape the immune tolerant microenvironment. Hormones, specific pathway inhibitors, cytokine blockers and modulation of immune cells can all be used to counter inflammatory responses. Mesenchymal stem cells are regarded as one of the most promising emerging therapies. Precise transplantation of these cells can alleviate inflammation-induced testicular injury (259) (Table 2 summarizes the clinical and experimental therapeutic approaches for various diseases).

**Table 2:** Integration of treatment approaches for different diseases.

Pathological mechanisms	Clinically ready Interventions	Experimental/pre-clinical therapies
Hypoxia, ROS accumulation, NLRP3 activation, pro-inflammatory cytokine (IL-1/6) elevation, ASA generation.	Microsurgical/laparoscopic varicocelectomy, basic oral antioxidants.	Ghrelin, Celecoxib, Dexamethasone, Chlorogenic acid, Minocycline, Mailuoshutong pill (targeting PI3K/Akt/mTOR).
Ischemia-reperfusion injury, ROS burst, apoptosis activation, autoimmune response triggered by BTB disruption.	Surgical detorsion and orchiopexy, orchiectomy	Adipose-Derived Stem Cell-Derived Exosomes(ADSC-Exos), Curcumin, Rapamycin (mTORC1 inhibitor), PNX-14, NMN, Chlorogenic acid.
Ascending infection, Th1/Th17 imbalance, ASA formation in seminal plasma.	Antibiotics (for ABP/CBP), NSAIDs, supportive drainage.	Lycium barbarum polysaccharides, DHA, Sulforaphane (targeting Nrf2/HO-1), Xialiqi capsules (targeting NLRP3/Caspase-1).
Viral internalization triggering macrophage pyroptosis; EAO characterized by Treg depletion and catastrophic loss of tolerance.	Supportive care, MuV vaccination, targeted antibiotics (if non-viral).	Exogenous testosterone, GSDMD pore-forming inhibitors, VD3-induced restoration of immune tolerance.
Virus-mediated receptor activation (e.g., ACE2), robust ISG response, sperm DNA fragmentation (SDF).	Vaccination (HPV), Ceftriaxone/Doxycycline (for bacterial STI), Hyaluronidase-based sperm washing (IALu).	Exogenous IFN-β, CLEC5A/DAP12 axis blockade, Interferon-ε.
Aryl hydrocarbon receptor (AhR) activation, ROS-mediated NF-κB upregulation, BTB tight junction degradation.	Strict removal from exposure sources.	Gum Arabic, Caffeic acid, Rapamycin, ZG7, Melatonin, AhR antagonists (Resveratrol).
Testicular immunosenescence, SASP expression, M1/M2 macrophage shift, mitochondrial DNA mutation.	Systemic support for anti-aging.	Melatonin, Icariin, NAD+ precursors, Mitochondria-targeted peptides (SS-31), TGF-β receptor inhibitors.
Abnormal adipokine profiles, gut-testis axis disruption, TLR4 activation.	Weight management, lifestyle/dietary shifts (Mediterranean diet).	Metformin, Resveratrol, Mesenchymal stem cell-derived exosomes (MSC-Exos).

Future studies will leverage single-cell sequencing and spatial transcriptomics to pinpoint patient-specific inflammatory and oxidative profiles. This molecular precision is essential for selecting candidates who will truly benefit from immunotherapy. The development of nanoparticle carriers for targeted delivery of anti-inflammatory or immunomodulatory agents to the testis through BTB could increase local drug concentrations. Large-scale, multicentre randomised controlled trials are essential to define the optimal dosing and combinations of immunomodulators. Data from these studies will serve as the foundation for establishing standardized clinical guidelines. These efforts may lessen the impact of inflammation on male fertility and offer hope to individuals facing reproductive challenges.

## 6 Clinical applicability and limitations

Conventional semen analysis alone is insufficient to fully assess male fertility. We advocate incorporating molecular and inflammatory markers (specifically SDF and cytokines like IL-6) into routine screening to facilitate the early diagnosis of immune-mediated infertility. For patients presenting with elevated ROS or anti-sperm antibodies, a combined anti-inflammatory and antioxidant regimen is recommended. Furthermore, lifestyle interventions such as the Mediterranean diet offer a cost-effective strategy to modulate systemic inflammation via the gut-testis axis.

However, current approaches still exhibit certain limitations: incorporating SDF and IL-6 testing into routine screening facilitates the early detection of immunological abnormalities. However, significant individual variability prevents the accurate clinical assessment of male fertility potential. The seminal plasma immune microenvironment is heavily confounded by factors like local conditions and ejaculation frequency.

Furthermore, lacking clear international guidelines for inflammatory intervention thresholds, current immunological indicators serve only as supplementary references rather than independent diagnostic tools. Although combined anti-inflammatory and antioxidant therapies or lifestyle interventions cost significantly less than assisted reproductive technology, a recent meta-analysis indicates that antioxidant therapy does not improve clinical pregnancy rates (260). Patients receiving blind empirical treatments without precise immunophenotyping risk missing their optimal reproductive window. Consequently, they often eventually require alternative conception methods, which exacerbates the financial burden and psychological stress on both partners.

Much of the data regarding the BTB and NLRP3 cited in this review derives from animal models without robust validation in humans. Current findings rely heavily on rodent models. However, the anatomical architecture of the human BTB and its immune cell proportions differ significantly from those in animals. Consequently, this divergent immune microenvironment explains why many drugs exhibit profound efficacy in animal models but fail in clinical trials. Furthermore, utilizing nano-targeted carriers to deliver immunomodulatory drugs to the male reproductive system presents severe ethical challenges. Any targeted therapeutic crossing the BTB could inflict unknown, irreversible damage on spermatozoa. Moreover, no long-term studies currently exist to guarantee the absolute developmental safety of subsequent offspring.

Although the testis is an immune organ, current systemically administered immunomodulators can effectively penetrate the BTB to reach target sites. Nevertheless, mitigating the associated systemic adverse effects remains an urgent clinical challenge. Despite these hurdles, I firmly believe that ongoing technological advancements will ultimately yield more targeted and efficacious therapies for patients with male infertility.

## Glossary

ABP: Acute bacterial prostatitis
ACE2: Angiotensin I-converting enzyme 2
ADSC-Exos: Adipose mesenchymal stromal cell-derived exosomes
AFB1: Aflatoxin B1
AFTs: Aflatoxins
AhR: Aryl hydrocarbon receptor
AOX: Antioxidant supplementation
APCs: Antigen-presenting cells
Arp3: Actin-regulatory protein 3
ASA: Antisperm antibodies
BES: Basal ectoplasmic specialization
BEB: Blood-epididymal barrier
BMI: Body mass index
BTB: Blood-testis barrier
CBP: Chronic bacterial prostatitis
CEB: Celecoxib
CGA: Chlorogenic acid
CLEC5A: C-type lectin domain family 5 member A
COVID-19: Coronavirus disease 2019
CP/CPPS: Chronic prostatitis/chronic pelvic pain syndrome
DAMPs: Danger-associated molecular patterns
DCs: Dendritic cells
DEX: Dexamethasone
DFI: DNA fragmentation index
DHA: Docosahexaenoic acid
EAP: Experimental autoimmune prostatitis
EAO: Autoimmune orchitis
ECM: Extracellular matrix
ED: Erectile dysfunction
Eps8: Actin-bundling protein 8
ER: Endoplasmic reticulum
EVs: Extracellular vesicles
FAK: Focal adhesion kinase
FasL: Fas ligand
GA: Gum Arabic/Acacia senegal
GAS6: Growth arrest specific 6
GJs: Gap junctions
GSDMD: Gasdermin D
HPV: Human papillomavirus
IALu: Hyaluronidase-based sperm washing
IDO: Indoleamine 2,3-dioxygenase
IFN: Interferon
iNOS: Inducible nitric oxide synthase
ISGs: Interferon-stimulated genes
LBP: Lycium barbarum polysaccharide
LCs: Leydig cells
MDA: Malondialdehyde
MLST: Mailuoshutong pill
MMP-9: Matrix metalloproteinase-9
MPS: Mononuclear phagocyte system
mTORC: Mammalian target of rapamycin complex
MuV: Mumps virus
NAG: Neutral α-glucosidase
NF-κB: Nuclear factor kappa B
NLRs: NOD-like receptors
NLRP3: NOD-like receptor family pyrin domain containing 3
NMN: Nicotinamide mononucleotide
NOD: Non-obese diabetic (mice)
NPs: Nanoplastics
PAR2: Protease-activated receptor 2
PD-1: Programmed death-1
PD-L1: Programmed death ligand 1
PFOA: Perfluorooctanoic acid
PFOS: Perfluorooctane sulfonic acid
PK2: Prokineticin 2
PKC: Protein kinase C
PNX-14: Phoenixin-14
PPARγ: Peroxisome proliferator-activated receptor gamma
PRR: Pattern recognition receptors
PSBP: Prostate steroid-binding protein
RACK1: Receptor for activated C kinase 1
ROS: Reactive oxygen species
rpS6: Ribosomal protein S6
SA: Sialic acids
SARS-CoV-2: Severe acute respiratory syndrome coronavirus 2
SCs: Sertoli cells
SDF: Sperm DNA fragmentation
SFN: Sulforaphane
SOD: Superoxide dismutase
sORP: Static oxidation–reduction potential
SOX2: SRY-box transcription factor 2
TAC: Total antioxidant capacity
Tc: Cytotoxic T cells (CD8^+^)
TGF-β: Transforming growth factor-beta
Th: Helper T cells (CD4^+^)
TJs: Tight junctions
TLR: Toll-like receptor
TMs: Testicular macrophages
TNF-α: Tumor necrosis factor-alpha
TT: Testicular torsion
VC: Varicocele
VD3: Vitamin D3
VMI: Varicocele-mediated infertility
Zfp637: Zinc finger protein 637
ZIKV: Zika virus.
